# Transcriptome and Proteome Profiling of Different Colored Rice Reveals Physiological Dynamics Involved in the Flavonoid Pathway

**DOI:** 10.3390/ijms20102463

**Published:** 2019-05-18

**Authors:** Xiaoqiong Chen, Yu Tao, Asif Ali, Zhenhua Zhuang, Daiming Guo, Qiaoling Guo, Asad Riaz, Hongyu Zhang, Peizhou Xu, Yongxiang Liao, Jing Wang, Changhui Sun, Quanju Xiang, Xianjun Wu

**Affiliations:** 1 Key Laboratory of Southwest Crop Genetic Resources and Genetic Improvement, Ministry of Education, Rice Research Institute, Sichuan Agricultural University, Chengdu 611130, China; xiaochenq777@126.com (X.C.); 18408211896@163.com (Y.T.); asifalikalas@foxmail.com (A.A.); daiming_guo@outlook.com (D.G.); mao18728448201@163.com (Q.G.); asad.riaz76@gmail.com (A.R.); zhanghysd@163.com (H.Z.); xpzhxj@163.com (P.X.); liaoyongxiang123@163.com (Y.L.); jingwang406@sicau.edu.cn (J.W.); sunhui0307@163.com (C.S.); 2 Chengdu Life Baseline Technology, Chengdu 610041, China; zhuangzhenhua@genebang.com; 3 College of Resources, Sichuan Agricultural University, Chengdu 611130, China; xiangquanju@163.com

**Keywords:** flavonoid biosynthesis, iTRAQ, transcriptome sequencing, red rice, black rice, *Oryza sativa* L.

## Abstract

Black and red rice are rich in both anthocyanin and proanthocyanin content, which belong to a large class of flavonoids derived from a group of phenolic secondary metabolites. However, the molecular pathways and mechanisms underlying the flavonoid biosynthetic pathway are far from clear. Therefore, this study was undertaken to gain insight into physiological factors that are involved in the flavonoid biosynthetic pathway in rice cultivars with red, black, and white colors. RNA sequencing of caryopsis and isobaric tags for relative and absolute quantification (iTRAQ) analyses have generated a nearly complete catalog of mRNA and expressed proteins in different colored rice cultivars. A total of 31,700 genes were identified, of which 3417, 329, and 227 genes were found specific for red, white, and black rice, respectively. A total of 13,996 unique peptides corresponding to 3916 proteins were detected in the proteomes of black, white, and red rice. Coexpression network analyses of differentially expressed genes (DEGs) and differentially expressed proteins (DEPs) among the different rice cultivars showed significant differences in photosynthesis and flavonoid biosynthesis pathways. Based on a differential enrichment analysis, 32 genes involved in the flavonoid biosynthesis pathway were detected, out of which only *CHI*, *F3H*, *ANS*, and *FLS* were detected by iTRAQ. Taken together, the results point to differences in flavonoid biosynthesis pathways among different colored rice cultivars, which may reflect differences in physiological functions. The differences in contents and types of flavonoids among the different colored rice cultivars are related to changes in base sequences of Os06G0162500, Os09G0455500, Os09G0455500, and Os10G0536400. Current findings expand and deepen our understanding of flavonoid biosynthesis and concurrently provides potential candidate genes for improving the nutritional qualities of rice.

## 1. Introduction

Asian cultivated rice (*Oryza sativa* L.) is an important global crop that feeds approximately half of the human population [1]. Rice is generally categorized based on caryopsis color into red, black, and white cultivars. It is well known that black and red rice are more nutritious than white rice. Additionally, in comparison to white rice, black and red rice are richer in secondary metabolites such as phenols and flavonoids. Studies suggest that pigmented rice has important biological activities including stronger antioxidant capacity, reduced cardiovascular disease risk, and prevention of cholesterol absorption [2,3,4,5]. Therefore, an understanding of the genetic and biochemical bases of metabolic functions among different pigmented rice cultivars will be greatly appreciated.

Flavonoids are widely distributed secondary metabolites with a range of metabolic functions in plants. Most pigmented rice cultivars are rich in flavonoids, which are derived from phenolic secondary metabolites [6]. The major flavonoids in black rice are anthocyanins, mainly consisting of cyanidin-3-O-glucoside and peonidin-3-O-glucoside, whereas red rice is rich in proanthocyanidins and flavan-3-ols oligomers, which have catechin as the main extension unit [7,8,9,10,11]. Significant efforts have been made to elucidate the biosynthetic pathway of flavonoids as well as their regulation by myeloblastosis (MYB) and basic helix-loop-helix (bHLH) transcription factors together with WD40 proteins [12,13]. These transcription factors belong to multigenic families encompassing 162 members in *Arabidopsis* and 167 members in rice, and several of them participate in regulation of flavonoid biosynthesis [14,15,16]. There are also other factors that affect the regulation of flavonoid biosynthesis, including light and sugar [17,18,19]. Additionally, several genes are involved in photosynthesis, but only some of these genes participate in the regulation of flavonoid biosynthesis; for example, among dicotyledonous species, flavone formation is primarily catalyzed by CYP93B enzymes [20]. However, there has been no systematic study to date that has assessed whether differential expression of transcription factors affects flavonoid biosynthesis and leads to different flavonoid products. Therefore, in the current study we performed an expression analysis of the transcription factors involved in flavonoid biosynthesis among different pigmented rice cultivars. 

High-throughput profiling of transcripts and proteins is an efficient method for deciphering the regulatory networks of functional genes that coordinately control complex biological processes [21]. Moreover, bottom-up profiling of transcripts and proteins, together with coexpression network analyses, are powerful approaches for interrogating biological processes (e.g., development) and constitutes an important aspect of systems biology. While transcriptional profiling is the method of choice for investigating development because of its low cost, interrogation of changes in protein profiles is also crucial, as proteins ultimately control biological processes. A combination of both the transcriptome and proteome is important for providing an accurate illustration of physiological events. Technological advances have made it increasingly possible to detect mRNA expression by using RNA sequencing (RNA-Seq) and to probe protein abundance using iTRAQ (isobaric tags for relative and absolute quantitation) [22]. Due to post-translational turnover and alternative translation efficiency, the integrated measurement and interpretation of changes in transcripts and protein abundance are mandatory for generating a complete inventory of gene networks [21]. Thus, joint analyses of multiomics data offer a more comprehensive view of specific biological processes by increasing our understanding of gene networks [23]. In an attempt to identify the pathways and physiological factors responsible for the differences in different pigmented rice cultivars, an integrative approach was used for profiling gene activity by RNA-Seq and iTRAQ. We compared the differences in abundance of mRNAs and proteins in the caryopses of different pigmented rice cultivars, and we found several key genes that were differentially expressed in the flavonoid biosynthesis pathways. We argue that the joint analysis of gene and protein expression data provides a comprehensive representation of the physiological factors that regulate flavonoid biosynthesis in different pigmented rice cultivars. This approach expands and deepens our understanding of flavonoid biosynthesis while concurrently providing potential candidate genes for improving the nutritional qualities of rice.

## 2. Results

### 2.1. Overview of the Transcriptome and Proteome

**Figure 1:**
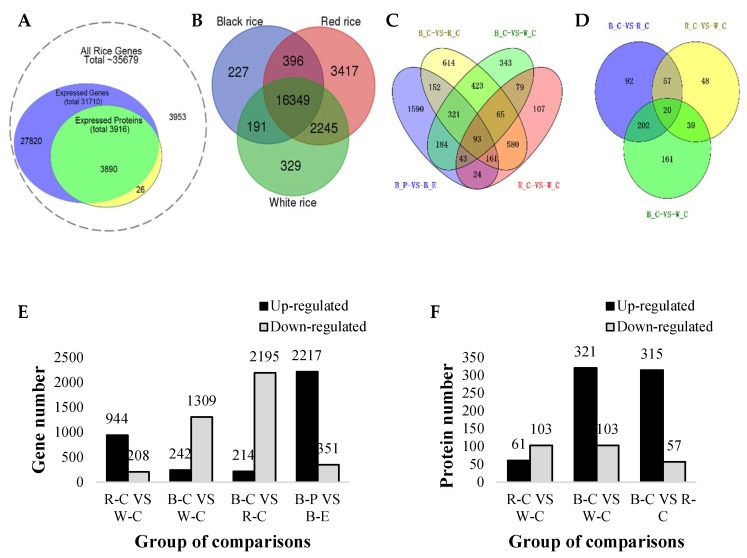
Comparisons of transcript and protein abundance in the different rice cultivars. (**A**) Congruency between the detected transcripts and proteins in the rice caryopses of the three different rice cultivars. (**B**) Venn diagram of commonly expressed and unique genes in the caryopses of black, red, and white rice. (**C**) Venn diagram of the differentially expressed genes (DEGs) in the caryopses of the different cultivars as well as the endosperm and pericarp of black rice. (**D**) Venn diagram of differentially expressed proteins (DEPs) in the caryopses of the different rice cultivars. (**E**) The number of DEGs and (**F**) proteins (DEPs) between the caryopses of the three cultivars (and the genes of the pericarp and endosperm of black rice). The black bars in (**E**) denote upregulated genes, whereas the gray bars represent downregulated genes. In (**F**), black bars denote upregulated proteins and grey bars represent downregulated proteins. W-C = white rice caryopsis, R-C = red rice caryopsis, B-C = black rice caryopsis, B-E = black rice endosperm, and B-P = black rice pericarp. The comparative analysis between black rice and red rice, red rice as control; comparative analysis between black rice and white rice, white rice as control; and comparative analysis between red rice and white rice, white rice as control.

Transcriptomes of caryopses of three rice cultivars were determined by RNA-Seq using a high-throughput Illumina platform. According to the RNA level line diagram (Supplementary Figure S1A), levels of gene expression in red rice were highest, followed by the pericarp of black, and then caryopses of black and white rice. A total of 31,700 genes were detected in 10 samples. Percentages of reads mapped to the genome ranged from 75.74%–84.73% (Supplementary Table S2). According to the congruence analysis between detected transcripts and expressed proteins, a total of 31,710 expressed genes and 3890 expressed proteins were detected (Figure 1A). Around 27,820 genes and 26 proteins were detected only in the transcriptome and proteome, respectively. Furthermore, 3953 rice genes were not detected in our study (Figure 1A). The number of specifically expressed genes was the highest in red rice (3417), followed by white rice (329), and then black rice (227) (Figure 1B). The three rice cultivars shared 16,349 genes in common (Figure 1B). We identified 13,996 unique peptides from 211,526 spectra, which corresponded to 3916 proteins in the six samples. The corresponding proteome was assessed and quantitatively catalogued using iTRAQ. The Mascot search algorithm was used to identify proteins. Robustness of the analysis was supported by multiple reaction monitoring (MRM) of six proteins from six samples (Supplementary Table S3). Principal component analysis (PCA) confirmed that the three different rice cultivars were genetically distinct based on gene expression (Supplementary Figure S1B). Additionally, the PCA showed relatively close distances between the replicate samples, whereas the distances between the different rice cultivars were significant (Supplementary Figure S1B). Furthermore, the pericarp and endosperm of the black rice were also genetically distinct.

### 2.2. Differentially Expressed Genes (DEGs) and Differentially Expressed Proteins (DEPs)

Differentially expressed genes (DEGs) were identified using NOIseq (log2FC (fold change) > 1 or log2FC < −1, probability > 0.8). When comparisons were made across the caryopses of the different rice cultivars, 614 uniquely expressed genes out of a total of 2409 DEGs (Supplementary Table S4) were discovered between red and black rice (Figure 1C). Similarly, between black and white rice, out of a total of 1551 DEGs, 343 genes were unique (Supplementary Table S4). DEG comparisons between the red and white caryopses showed that out of a total of 1152 genes 107 were unique DEGs (Supplementary Table S4).

Analysis and comparison of the proteomes of the different rice cultivars also revealed that 92 DEPs out of a total of 371 DEPs (Supplementary Table S5) were unique to black rice in comparison to red rice (Figure 1E). Similarly, 48 DEPs out of a total of 164 DEPs (Supplementary Table S5) were unique to red rice in comparison to white rice. Furthermore, 161 DEPs out of a total of 422 DEPs (Supplementary Table S5) were unique to black rice versus white rice (Figure 1E). Twenty DEPs were common to all rice cultivars (Figure 1D).

Comparisons of the different rice cultivars indicated that the numbers of upregulated DEGs were 944, 242, and 214 between red and white rice, black and white rice, and black and red rice, respectively (Figure 1D). In contrast, there were 208, 1309, and 2195 downregulated DEGs in the corresponding comparisons, respectively (Figure 1D). The number of upregulated genes was, thus, highest in red rice, followed by white rice and then black rice (Figure 1E).

DEPs were categorized based on 1.5-fold change (FC) in combination with a *p* value < 0.05. The numbers of upregulated DEPs were 61, 315, and 321 between red and white rice, black and red rice, and black and white rice, respectively (Figure 1F). In contrast, the numbers of downregulated DEPs were 103, 57, and 103 in the corresponding comparisons, respectively (Figure 1F).

### 2.3. Transcript Profiling of the Pericarp and Endosperm of Black Rice

The pericarp of black rice is pigmented because of the accumulation of a variety of anthocyanins, while the endosperm is colorless. Therefore, we analyzed the transcriptomes of the pericarp and endosperm in order to understand their role in color variation in rice. Comparisons of the endosperm and pericarp of black rice revealed 2568 DEGs, of which 1590 were unique. Ninety-three DEGs were common to all rice cultivars including the black rice pericarp vs. endosperm (Figure 1C). Furthermore, DEG analysis (Figure 1E) indicated that the number of upregulated genes in pericarp vs. endosperm (2217) was almost seven times that of the downregulated genes (351).

### 2.4. Gene Ontology (GO) Analysis of DEGs and DEPs in the Different Rice Cultivars

**Figure 2:**
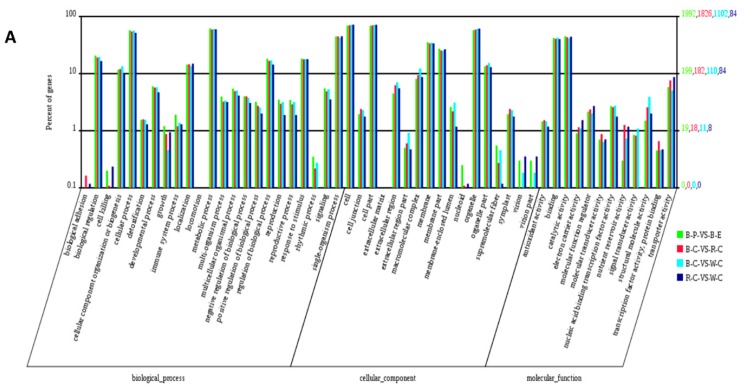

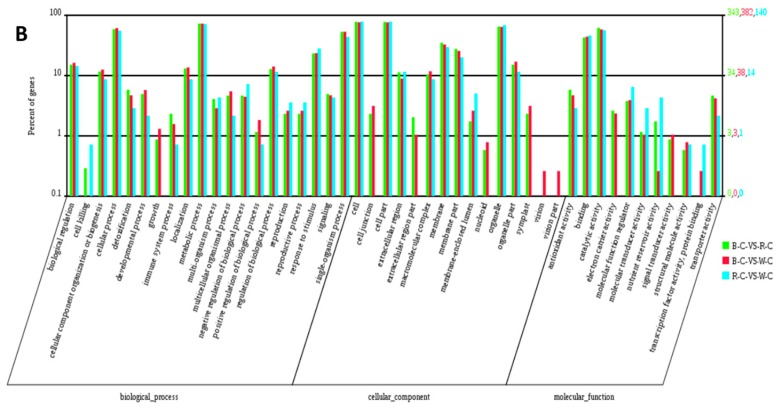
Gene ontology (GO) function depiction of the DEGs and DEPs by WEGO analysis. (**A**) Web Gene Ontology Annotation Plotting (WEGO) analysis of the different comparisons for the transcriptome, and (**B**) WEGO analysis of the different comparisons for the proteome. The abscissa represents each GO term. The left *Y*-axis represents the percentage of each term, whereas the right *Y*-axis represents the genes/proteins corresponding to each GO term. Different colored bars represent different comparisons.

To analyze the functions of DEPs and DEGs in different rice cultivars, gene ontology (GO) enrichment analyses were performed on four sets of DEGs (black rice pericarp vs. endosperm, black rice vs. red rice caryopsis, black rice vs. white rice caryopsis, and red rice vs. white rice caryopsis) and three sets of DEPs (black rice vs. red rice caryopsis, black rice vs. white rice caryopsis, and red rice vs. white rice caryopsis) based on the GO annotations (http://geneontology.org/). Figure 2 was drawn in Web Gene Ontology Annotation Plotting (BGI WEGO) (http://wego.genomics.org.cn/cgi-bin/wego/index.pl) to show the percentage of DEGs and DEPs in the three GO categories: biological process, cellular component, and molecular function (Figure 2; Supplementary Tables S6 and S7). DEGs involved in biological processes were distributed in cellular processes, metabolic processes, and single-organism processes and were highest in comparison to the other processes. Photosynthesis was particularly enriched (Supplementary Table S6), followed by metabolic pathways including flavonoid biosynthesis. Among cellular components, DEGs were highest in cells, cell parts, and organelle parts (Figure 2A). In the molecular functions category, DEGs were highest in binding and catalytic activity followed by transporter activity. A similar pattern was observed for DEPs in the GO term annotation (Figure 2B, Supplementary Table S7). In addition to the high proportion of GO terms associated with metabolic processes, we also recorded DEGs and DEPs enriched in the response to stimulus, immune response, and developmental processes. Results of the enrichment test for DEPs indicated that rice proteins were primarily related to cell components and metabolic processes (Figure 2B, Supplementary Table S7).

**Figure 3:**
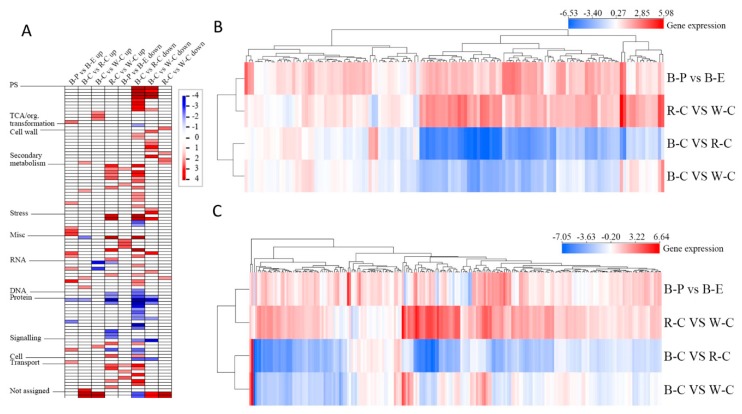
PageMan and cluster analyses of the DEGs in the different rice cultivars. (**A**) PageMan analysis for the different paired comparisons of DEGs between the rice cultivars (the term of significant enrichment is defined as *p* < 0.01); (**B**) expression profiles and cluster analysis of 211 DEGs in the photosynthesis pathway; and (**C**) expression profiles and cluster analysis of 132 genes in the flavonoid biosynthesis pathway.

To further assess differences among the different rice cultivars, we performed a PageMan analysis (*P* < 0.01 as the threshold) for different paired comparisons (Figure 3A). Results indicated significant differences in the photosynthesis pathway, secondary metabolism, abiotic and biotic stress responses, and other pathways. Downregulated enrichment in black rice was far greater than in red rice (Figure 3A). Based on annotation results of flavonoid-related genes in the rice database, we selected 211 genes involved in flavonoid biosynthesis and performed a hierarchical cluster analysis (Figure 3B). We also selected 132 genes that participated in flavonoid biosynthesis regulation in the photosynthesis pathway and performed a hierarchical cluster analysis (Figure 3C). Results showed that the number of upregulated genes in red rice was higher than black and white rice, whether directly or indirectly participating in flavonoid biosynthesis. Then, we further analyzed DEGs involved in biosynthesis of flavonoid components in an attempt to better understand the genetic background of the differential expression of flavonoid components among paired comparisons. Thirty-two genes showed significant differences, including 26 annotated genes and six unannotated genes. 

Importantly, these 32 genes mainly belonged to flavonoid biosynthesis and photosynthesis-related pathways (Table 1). Out of those genes, Os11G0599200 encoding UDP-GLYCOSYLTRANSFERASE 72B3, Os01G0850900 encoding the HEME-BINDING-LIKE protein At3g10130, Os06G0593800 encoding CROCETIN GLUCOSYLTRANSFERASE, Os02G0503100 encoding CYTOCHROME P450 71A1, Os09G0275400 encoding PREMNASPIRODIENE OXYGENASE, and Os04G0320700 encoding 7-DEOXYLOGANETIN GLUCOSYLTRANSFERASE were important. Furthermore, the expression level of Os01g0906450 (−7.01 FC level) was highest in black rice compared with red rice. In particular, the expression level of Os01G0372500 encoding LEUCOANTHOCYANIDIN DIOXYGENASE for anthocyanin synthesis was higher in black rice than in red rice (Table 1). Genotyping analysis of the 32 genes involved in flavonoid biosynthesis revealed that 232 single nucleotide polymorphisms (SNPs) existed between the pigmented and white rice cultivars. Further, SNPs were annotated by ANNOVAR software [24], and 51 synonymous mutations, 63 nonsynonymous mutations, 1 stoploss, and 2 stopgain mutations were found. Notably, GATK (Genome Analysis Toolkit, https://software.broadinstitute.org/gatk/) software revealed that four genes (Os06G0162500, Os09G0455500, Os09G0455500, and Os10G0536400) in the black and red rice cultivars existed in a heterozygous site, but they were located in a homozygous site in white rice (Supplementary Table S8).

We next analyzed the most significant differences in protein level abundance (Table 2). Results showed that some of the genes encoding proteins with significantly different expression levels showed inconsistent trends; for example, gene Os03G0367101 encoding FLAVONOID 3′,5′-HYDROXYLASE presented a significant difference in mRNA expression (Table 1), but it was not significantly different at the protein level. Gene Os01G0176000 encoding UDP-GLYCOSYLTRANSFERASE 73C6 presented significant differential expression at the protein level (Table 2) but not at the mRNA level. Eight genes with significant differences in their expression and protein abundance are given in Table S1.

### 2.5. Gene Coexpression Network Analysis

**Figure 4:**
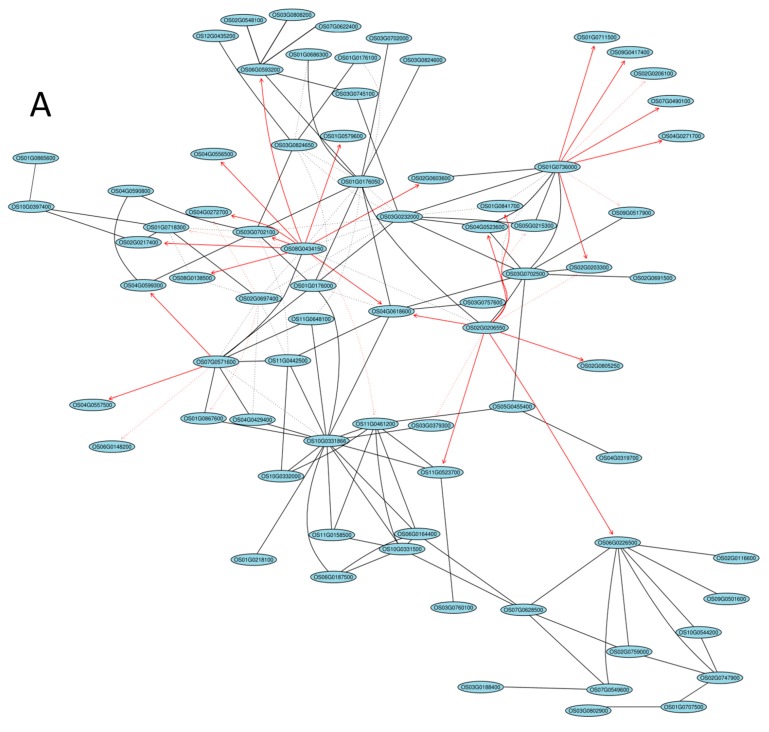

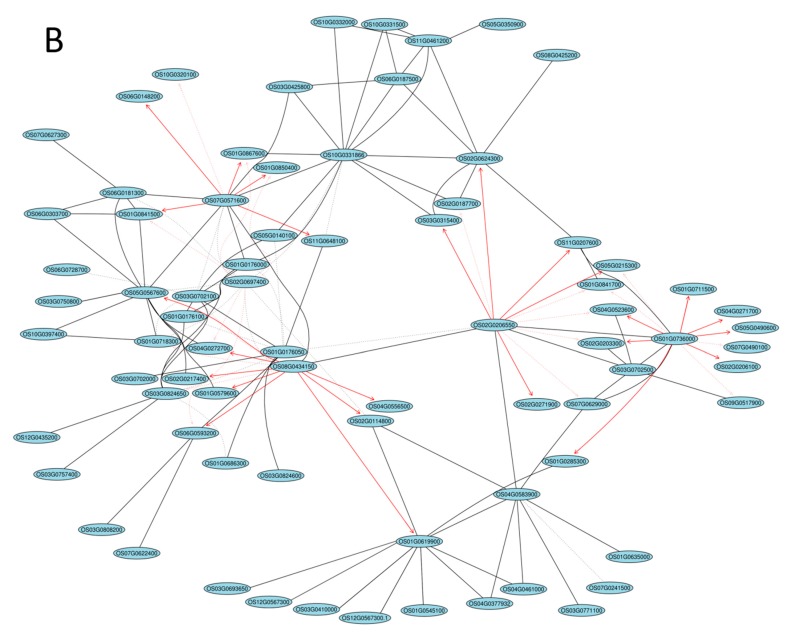
Network analysis between different pathways and flavonoid synthesis. (**A**) Basic helix-loop-helix (bHLH) and flavonoids; (**B**) Myeloblastosis (MYB) and flavonoids. The solid and dotted lines indicate positive and negative correlation coefficients, respectively, and the line intensity denotes their strength. Each straight arrow, from tail to head, indicates the interaction direction. A red color indicates that the Pearson’s correlation coefficient is greater than 0.8. The gene networks were obtained using the “GeneNet” method from the R package. The top 100 largest absolute correlations are displayed.

We performed regulatory network analysis by GeneNet to analyze and elucidate how flavonoid biosynthesis pathway components interact with other regulatory pathways such as photosynthesis, phenylalanine, and reactive oxygen species (ROS) metabolism pathways (Figure 4, Supplementary Table S9). Expression levels of bHLH and MYB transcription factors did not differ significantly in the different rice cultivars. However, a number of genes of the bHLH and MYB pathways were differentially expressed. The core genes Os08G0434150, Os10G0331866, and Os01G0736000 encoding bHLH played a key role in the regulation of networks associated with bHLH and the flavonoid biosynthesis pathway (Figure 4A). They probably directly or indirectly regulated the flavonoid biosynthesis pathway; for example, OS08G043415 regulates the flavonoid biosynthesis pathway via the Os04G0272700 encoding UDP-GLYCOSYLTRANSFERASE 92A1 and Os03G0702100 (hypothetical protein). The core genes Os08G0434150, Os01G0619900, and Os02G0206550 encoding MYB, which participated in the flavonoid biosynthesis pathway, also played a key role in regulatory networks involved in sugars, ROS metabolism, and photosynthesis pathways (Figure 4B). Peroxide and flavonoid regulation network core genes were OS01G0736000, Os02G0206550, and Os08G0434150 (Supplementary Figure S2A); while phenylalanine and flavonoid regulation network core genes were Os01G0736000, Os02G0206550, and Os07G0571600 (Supplementary Figure S2B). In terms of photosynthesis and the flavonoid regulation network, the core genes were Os08G0434150, Os01G0736000, and Os02G0206550 (Supplementary Figure S2C), whereas for sugar and the flavonoid regulation network, the core genes were Os07G0571600, Os02G0264700, and Os02G0206550 (Supplementary Figure S2D).

### 2.6. Correlation Analysis between the Proteome and Transcriptome of Different Rice Cultivars

**Figure 5:**
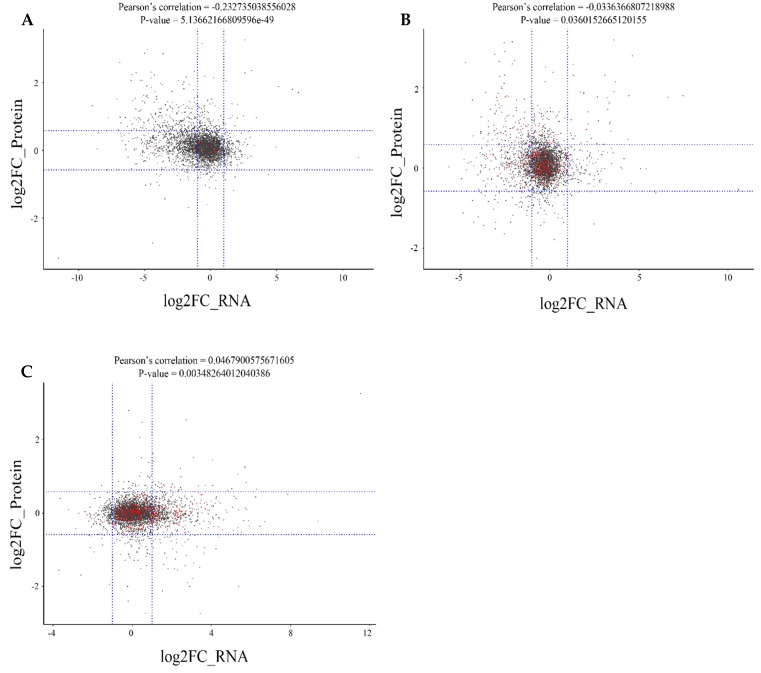
Correlation analysis between the expressed genes and proteins. (**A**) Analysis for black and red rice; (**B**) analysis for black and white rice; and (**C**) analysis for red and white rice. The red dot represents genes and proteins for flavonoid, photosynthesis, sugar, phenylalanine, peroxide, MYB, and bHLH transcriptome factors. The black dot represents the other genes and proteins. The blue dotted lines express the thresholds of differential expression (the fold-change of the RNA is log2|FC| > 1; the fold-change of a protein is log2|FC > 1.5 or FC < 0.667).

To explore the relationship between proteins and their cognate genes, we matched all expressed proteins with their cognate mRNAs among the three different pigmented rice cultivars (Figure 5, Supplementary Table S10). A weak, negative correlation was observed when black and red rice were compared (r value = −0.2327; Figure 5A) and when black and white rice were compared (r value = −0.0336; Figure 5B), whereas a weak, positive correlation was observed between red and white rice (r value = 0.0468; Figure 5C). For example, correlation analysis of transcripts vs. peptides between black and red rice revealed that a handful of mRNAs showed a tendency for downregulation, whereas the corresponding proteins showed upregulation, including flavonoid and flavonoid biosynthesis-related factors (Figure 5A). This indicated strong post-translational regulation. Analysis of all expressed genes with proteins between black and red rice showed a tendency for the expression of most of the expressed genes to be the same as the corresponding proteins, including those relating to flavonoid biosynthesis and transcription factors regulating flavonoid biosynthesis such as sugar, bHLH, MYB, and light (Figure 5A). An opposite scenario was observed between the expressed genes and proteins when black and white rice were compared (Figure 5B). Here, expression of all genes coincided with a similar pattern of protein presence. When red and white rice were compared, some mRNAs were downregulated, whereas corresponding proteins levels were not changed (Figure 5C). In general, there was weak consistency between gene expression and proteins.

Based on relative expression analysis, 204 genes/proteins that were significantly and differentially expressed in the different rice cultivars between transcript and protein analyses were selected (Supplementary Table S10). Fifty-six transcripts and proteins were consistently and differentially expressed between black and red rice, whereas 104 transcripts and their proteins were consistently and differentially expressed between black and white rice. Additionally, 164 transcripts and proteins were consistently and differentially expressed when red and white rice colored cultivars were compared. In contrast, 127, 62, and 15 genes were inconsistent between transcriptome and protein analyses for paired comparisons of the three rice cultivars (black vs. red, black vs. white, and red vs. white, respectively) such as Os01g0106400 encoding ISOFLAVONE REDUCTASE homolog of IRL, Os01g0124650 encoding BOWMAN-BIRK TYPE BRAN TRYPSIN INHIBITOR, and Os01g0228600 encoding HYDROXYPHENYLPYRUVATE REDUCTASE (Table 3). Furthermore, 21, 36, and 25 genes were consistent between transcript and protein analyses for the paired comparisons of the three rice cultivars (black vs. red, black vs. white, and red vs. white, respectively) including Os01g0372500 encoding LEUCOANTHOCYANIDIN DIOXYGENASE. Interestingly, the number of genes with inconsistent relationships between transcript and protein analyses was highest between black and red rice among the paired comparisons. This indicated the need for a joint analysis of the transcriptome and proteome.

### 2.7. Evaluation of Data Related to the Flavonoid Biosynthesis Pathway

**Figure 6:**
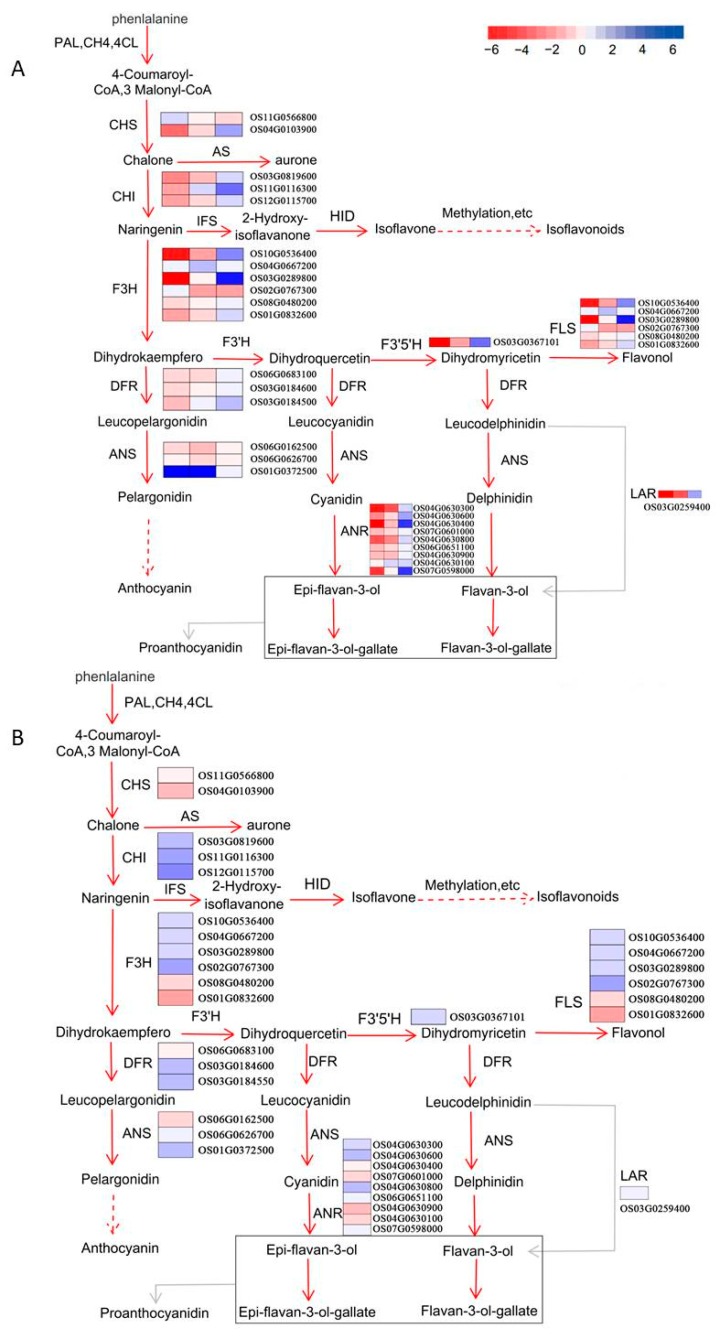
Pathway map visualizing the changes involved in genes, proteins, and metabolites for the flavonoid biosynthesis pathway among the three rice cultivars. (**A**) Pathway map for the three cultivars; and (**B**) pathway map for the pericarp and endosperm of black rice. ANR, anthocyaninidin reductase; ANS, anthocyanin synthase; AS, aurone synthase; C4H, cinnamate 4-hydroxylase; CHI, chalcone isomerase; 4CL, 4-coumarate coenzyme A ligase; CHR, chalcone reductase; F3H, flavanone 3-hydroxylase; F3′H, flavonoid 3-hydroxylase; F3′,5′H-hydroxylase; FLS, flavonol synthase; HID, 2-hydroxyisoflavanone dehydratase; IFS, 2-hydroxyisoflavanone synthase; LAR, leucoanthocyanidin reductase; PAL, L-phenylalanine ammonia-lyase; and TAL, L-tyrosine ammonia-lyase. Red font represents the key proteins identified by iTRAQ. Blue font represents the key metabolites.

Flavonoids are synthesized via the phenylpropanoid pathway (Figure 6) through the transformation of phenylalanine into 4-coumaroyl-CoA, which ultimately enters the flavonoid biosynthesis pathway. Based on enrichment analysis, we found 32 genes involved in the flavonoid biosynthesis pathway in rice. However, we only detected *CHALCONE ISOMERASE* (*CHI*), *FLAVANONE 3-HYDROXYLASE* (*F3H*), *ANTHOCYANIN SYNTHASE* (*ANS*), and *FLAVANOL SYNTHASE* (*FLS*) by iTRAQ (marked in red font in Figure 6), whereas others including *L-PHENYLALANINE AMMONIA-LYASE* (*PAL*), *CHALCONE SYNTHASE* (*CHS*), *DIHYDROFLAVONOL 4-REDUCTASE* (DFR), and *ANTHOCYANIN REDUCTASE* (ANR) were not detected. Naringenin, dihydrokaempferol, pelargonidin, dihydroquercetin, and cyanidin were the key metabolites identified by Liquid Chromatography/Mass Spectrometry (LC-MS). There were significant expressional differences in enzymes detected among the three different colored rice cultivars (Figure 6A). Interestingly, genes downstream in the flavonoid biosynthesis pathway differed most significantly among the different rice cultivars. For example, in black rice the principal DEGs were Os01G0832600, Os03G0184550, Os06G0626700, and Os06G0651100 encoding for *FLS*, DFR, ANS, and ANR, respectively. For red rice, the principal DEGs were Os03G0289800, Os06G0162500, and Os04G0630900 encoding FLS, ANS, and ANR, respectively. Genes involved in DFR presented low expression levels in red rice. In contrast, in white rice all genes in the flavonoid biosynthesis pathway presented rather lower expression levels than in black and red rice (Figure 6A). A significant difference in the pericarp and endosperm of black rice in the flavonoid synthesis pathway was also observed (Figure 6B). Many genes that contributed to differences in the different rice cultivars were also differentially expressed between the pericarp and endosperm of black rice, such as genes encoding for *F3H*, *ANS*, *FLS*, and *ANR*. This clearly suggested that genes downstream in the flavonoid synthesis pathway were more differentially expressed than those upstream in the flavonoid biosynthesis pathway.

**Figure 7:**
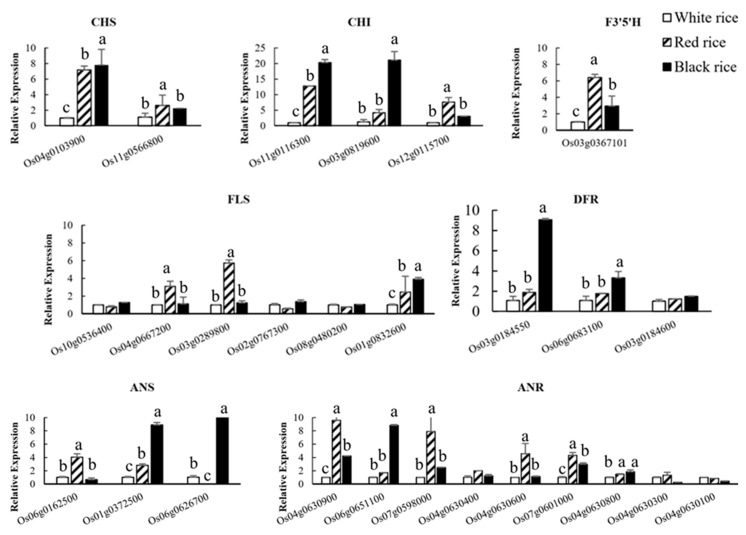
Quantitative analysis of the genes involved in the flavonoid biosynthesis pathway by qRT-PCR. Bars with identical superscripts letters denote relative expression of flavonoid biosynthesis pathway gene is not significantly different (*p* < 0.01) in different pigmented rice cultivars.

Quantitative expression analysis of genes involved in the flavonoid biosynthesis pathway corroborated transcriptomic data (Figure 7). This may be related to the stability of the enzymes. Additionally, most enzymes were regulated by a few genes, except for flavonoid 3′5′-hydroxylase, which was encoded by a single gene. For *CHS*, significantly higher expressions of Os04G0103900 in red and black rice were found compared to white rice. Similarly, all three genes involved in encoding *CHI* were significantly higher in red and black rice compared to white rice. Between red and black rice there were also significant differences in the expression of certain genes encoding for *CHI*, *F3′5′H*, *FLS*, *DFR*, *ANS*, and *ANR* (Figure 7).

## 3. Discussion

Our transcriptomic and proteomic study of different pigmented rice cultivars showed that there was substantial concordance between the proteins and transcripts. In fact, transcripts were detected for 99.34% of the proteins. Twenty-six proteins that were detected by proteomics analysis were not detected in the mRNA analysis, which was attributed to mRNA half-life and stability. Interestingly, 27,820 genes were only detected in the transcriptome, but there were no protein counterparts, which could be because they were transcription regulation factors. Thus, only a modest concordance (12.26%) was observed between the transcriptome and the proteome. Several differential transcriptomic and proteomic studies demonstrated a modest concordance between mRNA levels and proteins [24,25,26]. This discordance between mRNA and proteins can arise during the flow of genetic information from DNA to the ultimate phenotype. Furthermore, protein levels are influenced by translational and post-translational processes that result in a dynamic proteome [27].

The maximum number of upregulated DEGs was found when the caryopsis of red rice was compared with white rice, whereas a contrasting pattern was observed when black rice was compared with white rice. However, this trend was completely opposite when the proteomic data were analyzed. Upregulated DEPs were higher in black rice than white rice and lower in red rice than white rice. Many of these genes and proteins are involved in various metabolic pathways, including the biosynthesis of secondary metabolites, photosynthesis, and flavonoid biosynthesis. It is reasonable to speculate that various physiological factors, as well as the genes and proteins involved in the biosynthesis of flavonoid components, governed the observed metabolic differences and would have influenced DEGs and DEPs among the different rice cultivars. GO classification by WEGO analysis of DEGs and DEPs revealed enrichment in metabolic, photosynthetic, and flavonoid biosynthetic processes. Numerous studies suggested that transcription factors such as bHLH and MYB participated in the regulation of flavonoid biosynthesis genes [13,14,15,16]. Thus, transcription factors together with their interactions determine the activation as well as spatial and temporal expressions of structural genes in anthocyanin biosynthesis. A single enzyme can be synthesized by many genes; however, it is likely that differences between DEGs and DEPs are derived from the speed of mRNA synthesis and post-transcription regulation as well as other translational and post-translational mechanisms [12,28].

Significant differences in the different rice cultivars also existed in pathways associated with photosynthesis, secondary metabolism, and abiotic and biotic stress responses. Regulation of plant development and gene expression by light, an important environmental factor, is well known [29,30]. The elevation of anthocyanin pigments and *CHS* in response to light has been demonstrated in *Arabidopsis* seedlings [31]. Additionally, for metabolic enzymes and proteins involved in photosynthesis and anthocyanin biosynthesis, sugar is a common regulator [32]. It has been shown that sucrose induces anthocyanin accumulation and increases the expression of DFR, ANS, and LAR in rice and *Arabidopsis* [19,33].

It has also been demonstrated that flavonoids confer protection against stresses by reactive oxygen species (ROS) scavenging [34]. Thus, the synthesis of secondary metabolites, including flavonoids, in response to various environmental factors, such as strong light, ultraviolet radiation, high/low temperature, ozone, heavy metals, and drought, results in the generation of free radicals. One of the main functions of flavonoids is to attenuate effects caused by ROS [35,36]. In pigmented rice, the antioxidant capacity of the caryopsis is mainly derived from the pericarp, while very few differences exist in the endosperm compared to white rice [19]. This antioxidant effect is mainly derived from flavonoid synthesis. In dicotyledonous species, flavone formation is primarily catalyzed by cytochrome P450 enzymes [37]. Cytochrome P450 93G1 in rice has been shown to regulate flavone synthase [38]. Accumulations of epigallocatechin, quercetin, and other flavonoids are elevated by pathogen infection [39]. Therefore, we speculate that significant differences in flavonoid contents and types among the different pigmented rice cultivars are also the result of biotic factors.

Genotypic analysis of 32 genes involved in the flavonoid biosynthesis pathway revealed 232 SNPs in four genes in the different rice cultivars. It is reasonable to speculate that color and the high expression of flavonoid components are derived from these genes. Additionally, base changes in these four genes (Os06G0162500, Os09G0455500, Os09G0455500, and Os10G0536400) directly influenced the gene expression of the flavonoid biosynthesis pathway, leading to high contents of flavonoid compounds in pigmented rice in contrast to white rice, as these are key genes in the flavonoid biosynthesis pathway. For example, one SNP was located on chromosome 12 with coordinates of 768036, which was annotated with a nonsynonymous mutation. In white rice, its genotype was pure A, while in pigmented rice, its genotype was heterozygous GA. According to the qRT-PCR experiment, the corresponding *FLS* gene (Os12G0115700) expressed in pigmented rice was significantly higher than that in white rice. This suggests that nonsynonymous mutation may indeed affect the expression of key genes. However, further study is required to conclusively demonstrate that increased flavonoid contents are directly derived from SNPs in these genes.

Transcriptional regulation of structural genes for the flavonoid biosynthesis pathway is controlled by MYB and bHLH transcription factors as well as WD40 proteins [40]. Coexpression network analyses revealed that these transcriptional factors also regulated flavonoid biosynthesis by interacting with other pathways such as photosynthesis, sugar synthesis, and peroxidases. It has been reported that Rc encodes a bHLH protein involved in red caryopsis production, and a 14-bp deletion in Rc changes the caryopsis color to white [41]. In maize, different tissue-specific patterns of anthocyanin accumulation are derived from divergent controlling elements such as promoters and untranslated regions (UTRs) [42,43]. In morning glories *Ipomoea purpurea* and *Ipomoea tricolor*, a mutant of bHLH2 and *IVS* (*IVORY SEED*) affects the biosynthesis of anthocyanins and the accumulation of proanthocyanins [44]. However, there is no report on whether the contents and types of flavonoids, and coloration differences among different rice cultivars, are related to the expression levels of transcription factors. Our study showed that transcription factors did not differ significantly in expression among the different rice cultivars, whereas the downstream genes involved in the flavonoid biosynthesis pathway showed significant differences. This corroborates with an earlier report by Chen [19]. Therefore, it is reasonable to conclude that while transcription factors regulate flavonoid biosynthesis, the differences in contents and types of flavonoids among the different pigmented rice cultivars are due to changes in sequence differences, including the promoter region, UTR, and conserved domain of key genes involved in flavonoid biosynthesis.

Anthocyanins, proanthocyanins, and other flavonoid components are produced via the flavonoid biosynthesis pathway, and the majority of regulatory genes in rice have been identified by gene cloning and homology analyses [45,46,47,48]. A comparative study between different pigmented cultivars of the same species revealed that upstream genes (*CHS* and *CHI*) of the flavonoid biosynthesis pathway are expressed at similar levels in cauliflower and rice. However, downstream genes (*F3′H*, *DFR*, *ANS*, and *LAR*) of this pathway exhibit significantly different expression levels [4,49]. *AN1* and *AN4*, the genes encoding MYB transcription factors, activate the bHLH transcription factor of *AN1* in *Petunia* [50]. *Pr* and bHLH1 coordinately regulate several transcripts of anthocyanin late pathway genes to control pigment accumulation in cauliflower [49]. Coloration mechanisms of a plant depend on three different types of proteins, namely a color-producing protein, an anthocyanin biosynthesis activator, and a tissue-specific pigmentation protein [51,52]. Purple or black are related to the rearrangement in the promoter region of *Kala1*, whereas a red pericarp is produced by bHLH and DFR, and a brown pericarp is formed without the participation of DFR [41,47]. This study corroborates the results of earlier studies [4,49]. Based on gene expression validation by qRT-PCR, we speculated that the primary reason for the difference in flavonoid composition between black rice and red rice was related to DEGs in the flavonoid pathway. For example, three anthocyanin synthetase genes were detected. Comparisons of the different cultivars showed that one particular gene was highly expressed in black rice, while the expression of genes associated with anthocyanin synthesis in red rice was relatively low. This was why anthocyanin content in black rice was significantly higher than in red rice. In addition, proanthocyanidin content in red rice was higher than in black rice. Enrichment analysis indicated that one of the genes involved in proanthocyanidin synthesis was significantly enriched in red rice in comparison to black rice. Further analysis revealed that several genes were associated with each enzyme, and one or two genes with particularly high expression levels were present in different colored caryopses. Thus, differences existed between major gene effects and minor gene effects, and differences between black rice and red rice could mainly be attributed to differences in major genes. This further confirms transcriptome analysis results, that the differences in flavonoid compositions between red rice and black rice are due to differences in major genes.

Furthermore, this study revealed a novel mechanism based on physiological dynamics, in which differences in coloration, flavonoid content, and type among the different rice cultivars are related to differential expression levels of the main structural genes and a few minor effect genes involved in flavonoid biosynthesis. This then leads to differences in the expression of flavonoids such as anthocyanin, catechin, and quercetin.

In conclusion, this integrated transcriptomic and proteomic analysis of different rice cultivars indicates the existence of important and dynamic physiological factors that govern flavonoid biosynthesis. It offers a foundation for the breeding of elite rice cultivars with enhanced natural and biologically active compounds for humans.

## 4. Materials and Methods

### 4.1. Plant Material

The following indica rice (*O. sativa*) lines were used for the transcriptomic and proteomic profiling: these lines were breed by Sichuan, China, the red rice cultivar “Hongnuo,” the black rice cultivar “Lianjian33,” and the white rice cultivar “Yixiang B” (Supplementary Figure S3). Rice plants were cultivated in a paddy field from April to September 2016 in Wenjiang District (latitude 30°429 N, longitude 103°509 E, altitude 539.3 m), Chengdu City, Sichuan Province, China. For each sample, at least 50 plants were cultivated, and two independent biological replicates were processed. Caryopsis of all cultivars were collected 5 days after flowering for RNA extraction and iTRAQ protein analysis. In addition, pericarp and endosperm from black rice were collected, also at 5 days after flowering, for RNA extraction.

### 4.2. RNA Isolation and Library Preparation for Transcriptome Analysis

Total RNA was extracted from the caryopsis (along with the pericarp and endosperm of black rice) using TRIzol reagent (Invitrogen, Carlsbad, CA, USA) according to the manufacturer’s instructions and the extraction method of Kim [53]. The library was prepared as follows: for RNA sample preparation, we used 3 µg RNA per sample as input. NEBNext^®^ Ultra™ RNA Library Prep Kit for Illumina^®^ (NEB, Ipswich, MA, USA) was used for sequencing libraries following the manufacturer’s protocol, and index codes were added to attribute each sample sequence. Magnetic beads of poly-T oligo-attached were used to purify the mRNA from total RNA. cDNA strands were first synthesized via random hexamer primer and MuLV Reverse Transcriptase and second via DNA polymerase I and RNase H. An AMPure XP system (Beckman Coulter, Beverly, Kraemer Boulevard Brea, CA, USA) was used to purify the fragments, which were used to increase sections of cDNA fragments by 150–200 bp. After that, 3 µL USER (Uracil-specific Excision Reagent, NEB) enzymes was added to adaptor-ligated cDNA for 15 min at 37 °C and followed by 5 min at 95 °C prior to PCR. Then, PCR was performed with universal PCR primers, index(X) primer, and with high-fidelity DNA polymerase. The quality of purified PCR products was checked on an Aglient Bioanalyzer 2100 system. Library preparations were sequenced on the Illumina HiSeq X Ten platform, and 150-bp paired-end reads were generated. The raw files (FASTQ format) can be accessed from the NCBI Sequence Read Archive (SRA) platform (https://trace.ncbi.nlm.nih.gov/Traces/sra/sra.cgi) under the accession number SRA719903.

### 4.3. Transcriptome Analysis

RNA-Seq raw reads were quality controlled and cleaned by SOAPnuke software (http://www.seq500.com/uploadfile/SOAPnuke.zip) to remove low-quality reads, contaminated reads, and reads with adapters. The resulting clean reads were then aligned to the rice IRGSP database (http://rice.plantbiology.msu.edu/pub/data/Eukaryotic_Projects/o_sativa/annotation_dbs/pseudomolecules/version_7.0/) and matched with rice genes by HISAT2 [53]. After the number of reads mapped to each gene was counted, the FPKM (fragments per kilobase per million fragments) method was used for normalization through (RNA-Seq by Expectation Maximization) RSEM [54] (https://deweylab.github.io/RSEM/), and the lowly expressed genes (FPKM < 1) were filtered in each sample. NOIseq [55] was employed to calculate the log 2-fold change (log2FC) and probability value for each gene in every comparison, and strict criterion were used (log2FC > 1 or log2FC < −1, probability > 0.8). Mapman software was used for PageMan analysis [56], and we defined the threshold of significant enrichment term as *p* < 0.01. ANNOVAR software was used for SNP functional annotation with the parameter: protocol refGene—operation g.

### 4.4. Protein Preparation

Proteins were extracted from mature caryopses (5 g), as previously described [56]. Briefly, caryopses were ground in liquid nitrogen and homogenized using buffer A (500 mM Tris-HCl/L pH 7.5, 50 mM EDTA, 100 mM KCl, 700 mM sucrose, 2% β-mercaptoethanol, 1× protease inhibitor, and 1 mmol/L phenylmethylsulfonyl fluoride (PMSF)) with 180 W ultrasonication for 10 min on ice. Then, buffer B (Tris-HCl pH 7.5 saturated phenols) was added to the sample at a rate of equal volume, placed on ice for 3 min to get a homogenized mixture, and subjected to centrifugation (15,000× *g*, 10 min). Protein extraction was done from the upper organic phase using buffer A and followed by chilled buffer C for protein precipitation at −20 °C overnight. Then, the sample was subjected to centrifugation, followed by washing thrice using chilled buffer C and two times with chilled acetone. 

Then, the protein samples were suspended in solubilizing buffer (7 M urea, 2 M thiourea, 4% CHAPS, 40 mM Tris-HCl, pH 8.5, 1 mM PMSF, and 2 mM EDTA) followed by sonication at 4 °C (pulse-on 2 s, pulse-off 3 s, power 180 W). Then, the samples were centrifuged at 20,000× *g* for 30 min and condensed in dithiothreitol (DTT; 10mM) for 60 min at 55 °C. After reduction, the samples were alkylated by iodoacetate (55 mM) for 60 min in the dark, precipitated in chilled acetone (4 × volume) at −20 °C overnight, followed by centrifugation at 20,000× *g* for 30 min at 4 °C. The pellet was dissolved in 400 μL of 0.5 M TEAB (Applied Biosystems, Milan, Italy) and sonicated at 4 °C for 3 min. The dissolved pallet was subjected to centrifugation at 20,000× *g* for 30 min at 4°C, the supernatant was collected, and protein concentration was determined by the Bradford assay.

### 4.5. iTRAQ Labeling, Strong Sation Exchnge Chromatography (SCX) Fractionation, and Liquid Chromatography-Electrospray Ionization-Tandem Mass Spectrometry (LC-ESI-MS/MS) Analysis

Proteins (100 μg) of each sample were digested using Trypsin Gold (Promega, https://www.promega.com.cn/, Germany) at 37 °C for 16 h (protein: trypsin = 30:1). Then, the peptides were dried by vacuum centrifugation, reconstituted in 0.5 M TEAB, and processed with 6-plex iTRAQ labeling reagent according to the manufacturer’s protocol. The samples were labeled by the iTRAQ tags as follows: black rice 1 (114), black rice 2 (115), red rice 1 (117), red rice 2 (118), white rice 1 (119), and white rice 2 (121). After the peptides were labeled with isobaric tags and incubated at room temperature for 2 h, they were pooled and subsequently dried by vacuum centrifugation. Chromatography was performed by an Ultramate 3000 nano ultra-performance (UP)LC with a C18 column, and tandem mass spectrometry (MS/MS) was performed by a Q Exactive Orbitrap mass spectrometer (Thermo Fisher Scientific, Germany), as previously described [57]. iTRAQ proteomics analysis was performed twice for all samples.

### 4.6. Proteome Data Analysis

The raw data files generated by Orbitrap were converted to mascot generic format (MGF) by Proteome Discoverer 1.2 (Thermo Fisher Scientific). These MGF files were subjected to search against proteins in rice (http://rice.plantbiology.msu.edu/pub/data/Eukaryotic_Projects/o_sativa/annotation_dbs/pseudomolecules/version_7.0/all.dir/all.pep) by Mascot v2.3.02 (Matrix Science). To identify and quantify the proteins in the rice grains, the following parameters were used: iTRAQ 8-plex; enzyme: trypsin; fixed modification: carbamidomethyl (C), iTRAQ 8-plex (N-term), and iTRAQ 8-plex (K); variable modifications: dioxidation (M), oxidation (M), and iTRAQ 8-plex (Y); mass values: monoisotopic; peptide mass tolerance: ± 15 ppm; fragment mass tolerance: ± 20 mmu; max missed cleavages: 1; and charge states of peptides: +2 and +3. 

The decoy checkbox was selected in Mascot for an automatic decoy database search, which generated a random sequence database. The random sequence database and real database were tested to get raw spectra. Peptides with a significant score of ≥20 at the 99% confidence interval, for Mascot probability analysis greater than “identity”, were counted as identified, which reduced false peptide probability. Each confident protein identification was associated with at least one unique peptide. The iTRAQ proteomics analysis was performed twice, and differentially expressed proteins (DEPs) were identified with a fold change of >1.5 (*p* < 0.05) or <0.67 (*p* < 0.05).

To analyze the potential functions of the proteins, we first reannotated the rice proteins. Briefly, rice proteins were mapped to multiple public databases, including the NCBI nonredundant (NR), Swiss-Prot/UniProt, Gene Ontology (GO), and Kyoto Encyclopedia of Genes and Genomes (KEGG) databases. Using all the proteins as background, we used the numbers of differentially expressed proteins (DEPs) to calculate the *p*-value and *Q*-value, which represented the significance of the enriched GO terms/KEGG pathways and the false discovery rate (FDR), respectively. The *p*-values were calculated by Fisher’s exact test, and the *Q*-values were calculated by an R package named “q-value” [58]. The threshold of significance was defined as *FDR* < 0.05.

### 4.7. Gene Coexpression Network Analysis

The “GeneNet” package was used to construct gene coexpression networks [59]. This method used partial correlation to calculate the link between two genes and had the advantage of not requiring any parameters (with the exception of the correlation threshold used to select the most relevant edges). Coexpression network analyses were constructed for the regulation between photosynthesis and flavonoids, between sugars and flavonoids, between phenylalanine and flavonoids, between peroxide and flavonoids, between MYB and flavonoids, and between bHLH and flavonoids. We displayed the top 150 largest absolute correlations, and the graph was generated using the “ggplot2” package [60].

### 4.8. qRT-PCR Analysis

qRT-PCR was performed using a qTOWER^3^G Real-Time System (Analytik-Jena, Germany), following the manufacturer’s instructions. All reactions were performed with a ChamQ Universal SYBR qPCR Master Mix (Vazyme, Nanjing, China) according to the manufacturer’s protocol. The housekeeping gene GAPDH was used as the internal control. The sequences of the primers are indicated in Supplementary Table S11.

### 4.9. Statistical Analysis

All transcriptional and proteomic samples were designed for two biological replicates. To identify the DEGs, NOIseq was used to calculate the log 2-fold change (log2FC) and probability for each gene in every comparison, and a strict criterion was used (log2FC > 1 or log2FC < −1, probability > 0.8). We performed iTRAQ proteomics analyses four times, and the DEPs were identified if the ratio was >1.5 and the *p*-value (*t*-test) was <0.05. Fisher’s exact test and the *Q*-values were calculated by an R package in the GO and pathway enrichment analysis, and an FDR less than 0.05 constituted the significance threshold.

## Figures and Tables

**Table 1:** Significantly different genes based on fold-change levels for the three rice cultivars.

Gene ID	log2FC(B-C/R-C)	log2FC(B-C/W-C)	log2FC(R-C/W-C)	Description
Os11G0530650	−5.840043541	-	5.103077947	-
Os11G0599200	−5.734374274	-	3.273644684	UDP-glycosyltransferase 72B3
Os02G0188000	−4.831826158	-	-	cinnamate beta-D-glucosyltransferase
Os01G0179600	−5.138751765	-	2.797789001	crocetin glucosyltransferase, chloroplastic
Os03G0367150	−5.252202169	−2.676197753	2.576004415	-
Os01G0850900	-	-	2.930368899	heme-binding-like protein At3g10130, chloroplastic
Os08G0434100	−5.040311748	−3.361280923	-	extracellular ribonuclease LE
Os10G0320100	-	2.681785408	3.706832427	flavonoid 3′-monooxygenase
Os06G0608700	−4.855820887	−3.156281607	-	fructose-bisphosphate aldolase, cytoplasmic isozyme 1
Os01G0638000	−5.070762648	-	-	anthocyanin 3′-O-beta-glucosyltransferase
Os06G0593800	−5.097684878	-	2.81431663	crocetin glucosyltransferase, chloroplastic
Os11G0116300	-	-	3.4782357	probable chalcone--flavonone isomerase 3
Os02G0503100	−6.152067498	-	3.052531824	cytochrome P450 71A1
Os09G0275400	−5.060047384	-	5.773166236	premnaspirodiene oxygenase
Os08G0547900	-	-	4.285402219	cytochrome P450 76M5-like
Os03G0757000	-	-	5.039052777	UDP-glycosyltransferase 83A1
Os02G0697400	-	3.901446457	-	probable 4-coumarate--CoA ligase 2
Os11G0530600	-	4.641474522	6.273915733	-
Os01G0372500	6.196397213	6.63840176	-	leucoanthocyanidin dioxygenase
Os03G0757200	−6.432291338	-	4.133633023	-
Os03G0819600	-	−1.607364477	-	chalcone--flavonone isomerase
Os06G0165800	-	−1.984442653	-	caffeoyl-CoA O-methyltransferase 1
Os02G0704000	−6.716093942	-	4.661646158	zeaxanthin 7,8(7′,8′)-cleavage dioxygenase, chromoplastic
Os03G0367101	−5.912116705	-	3.786828232	flavonoid 3′,5′-hydroxylase 1
Os09G0441400	−4.957665163	-	-	cytochrome P450 71A1
Os02G0207400	-	-	2.489120349	UDP-glycosyltransferase 73C6-like
Os04G0320700	−6.380423744	-	4.443988873	7-deoxyloganetin glucosyltransferase
Os07G0503300	−6.050107771	−4.496425826	-	anthocyanidin 3-O-glucosyltransferase 2
Os01G0906450	−7.007961655	−7.047123912	-	-
Os06G0256500	-	−1.379661054	-	glucose-6-phosphate isomerase, cytosolic B
Os06G0288300	−6.14198546	-	4.766118558	UDP-glycosyltransferase 708A6
Os10G0320201	-	-	4.19440866	-

**Table 2:** Significantly different proteins based on fold-change levels for the three rice cultivars.

Protein ID	Mean Ratio B-C vs. R-C	Mean Ratio B-C vs. W-C	Mean Ratio R-C vs. W-C	Function
Os04G0206500	-	-	0.83	crocetin glucosyltransferase 2
Os01G0372500	3.48	3.38	-	leucoanthocyanidin dioxygenase
Os04G0662600	2.32	2.4	-	naringenin,2-oxoglutarate 3-dioxygenase
Os05G0527100	2.58	2.79	-	UDP-glycosyltransferase 88F3-like
Os03G0819600	1.54	1.31	-	chalcone--flavonone isomerase
Os09G0518000	1.39	1.49	-	crocetin glucosyltransferase 2
Os01G0686300	-	1.64	1.37	cinnamate beta-D-glucosyltransferase
Os01G0850900	1.33	1.35	-	heme-binding-like protein At3g10130, chloroplastic
OS11G0384789	-	-	0.83	-
Os02G0816600	-	0.66	0.56	uncharacterized protein sll0005
Os07G0510500	4.54	3.62	0.8	anthocyanidin 3-O-glucosyltransferase 2
Os08G0434100	-	1.35	1.44	extracellular ribonuclease LE
Os10G0320100	3.04	3.42	-	flavonoid 3′-monooxygenase
Os01G0638000	1.68	1.46	0.87	anthocyanin 3′-O-beta-glucosyltransferase
Os01G0805400	0.56	1.53	2.67	UDP-glycosyltransferase 87A1
Os04G0523600	0.87	-	1.5	UDP-glycosyltransferase 73C7
Os12G0115700	2.55	2.32	-	probable chalcone--flavonone isomerase 3
Os06G0192100	2.36	2.05	-	anthocyanidin 3-O-glucosyltransferase
Os04G0206700	0.76	-	1.42	UDP-glycosyltransferase 74F2
Os07G0503500	2.47	2.3	-	anthocyanidin 3-O-glucosyltransferase 2
Os01G0176000	5.05	2.06	0.46	UDP-glycosyltransferase 73C6
Os06G0289900	1.32	-	0.77	UDP-glycosyltransferase 708A6
Os03G0693600	-	1.4	1.38	indole-3-acetate beta-glucosyltransferase
Os06G0256500	-	-	0.78	glucose-6-phosphate isomerase, cytosolic B
Os06G0282000	-	-	1.26	UDP-glycosyltransferase 89B2
Os11G0530600	3.86	6.95	1.57	-
Os05G0133100	0.82	-	-	nitrogen regulatory protein P-II homolog
Os03G0808200	-	-	1.14	UDP-glucose flavonoid 3-O-glucosyltransferase 7
Os03G0776000	-	1.27	1.24	glucose-6-phosphate isomerase, cytosolic A
Os08G0547951	0.37	-	2.73	-
Os08G0174300	-	-	0.78	anthocyanin 5-aromatic acyltransferase

**Table 3:** Comparison of mRNA and protein expression levels.

Gene ID	BR_RNA	BR_PEP	BW_RNA	BW_PEP	RW_RNA	RW_PEP	Description
Os01G0106400	−5.30735	0.678072	-	-	-	-	isoflavone reductase homolog IRL
Os01G0124650	−6.88264	2.594549	−4.70223	2.634593	-	-	Bowman-Birk type bran trypsin inhibitor
Os01G0127600	-	-	-	-	5.728406	1.244887059	Bowman-Birk type bran trypsin inhibitor
Os01G0180000	-	-	−1.34699	−0.73697	-	-	leucine-rich repeat extensin-like protein 3
Os01G0228600	−2.30296	0.871844	-	-	-	-	hydroxyphenylpyruvate reductase
Os01G0317800	-	-	−1.58025	0.887525	-	-	caffeoylshikimate esterase
Os01G0372500	6.196397	1.799087	6.638402	1.757023	-	-	leucoanthocyanidin dioxygenase

Note: BR_RNA represents a comparison of the mRNA levels between black and red rice; BR_PEP represents a comparison of the protein levels between black and red rice; BW_RNA represents a comparison of the mRNA levels between black and white rice; BW_PEP represents a comparison of the protein level between black and white rice; RW_RNA represents a comparison of the mRNA levels between red and white rice; and RW_PEP represents a comparison of the protein level between and red and white rice.

## Supplementary Materials

Supplementary materials can be found at https://www.mdpi.com/1422-0067/20/10/2463/s1, Figure S1: Transcriptome analysis of samples (A) the mRNA level line diagram; (B) PCA analysis. Table S1. Significantly different genes based on fold-change levels for the three rice cultivars. Table S2: Overview of transcriptome quality for the caryopses of the three rice cultivars and the pericarp and endosperm of black rice. Table S3: All identified proteins in three cultivars of rice and basic statistics. Table S4: Differentially expressed genes in three cultivars of rice. Table S5: Differentially expressed proteins in three cultivars of rice. Table S6: The classification of WEGO for all DEGs. Table S7: The classification of WEGO for all DEPs. Table S8: Genotyping of 32 related genes in the flavonoid pathway in three cultivars of rice. Table S9: Significant difference genes/proteins in both RNA-Seq and iTRAQ for coexpression analysis. Figure S2: Gene coexpression network analysis. (A) The peroxide and flavonoid regulation network of the core gene analysis; (B) the phenylalanine and flavonoid regulation network of the core gene analysis; (C) photosynthesis and flavonoid regulation network of the core gene analysis; and (D) sugar and flavonoid regulation network of the core gene analysis. Table S10: List of flavonoid synthesis related genes for correlation between proteins and genes. Figure S3: Different pigmented rice cultivars used in this study. Table S11: List of RT-PCR primers for flavonoid biosynthesis pathway genes.
