# Supplementary material for: Transcriptome and Proteome Profiling of Different Colored Rice Reveals Physiological Dynamics Involved in the Flavonoid Pathway
Source: Int J Mol Sci. 2019 May 18;20(10):2463. doi: 10.3390/ijms20102463 (PMC6566916; doi:10.3390/ijms20102463)
Supplement: Supplementary file 1 [file ijms-20-02463-s001.zip › ijms-496936-proof done-supplementary/Figure S1.pptx]

## Slide 1
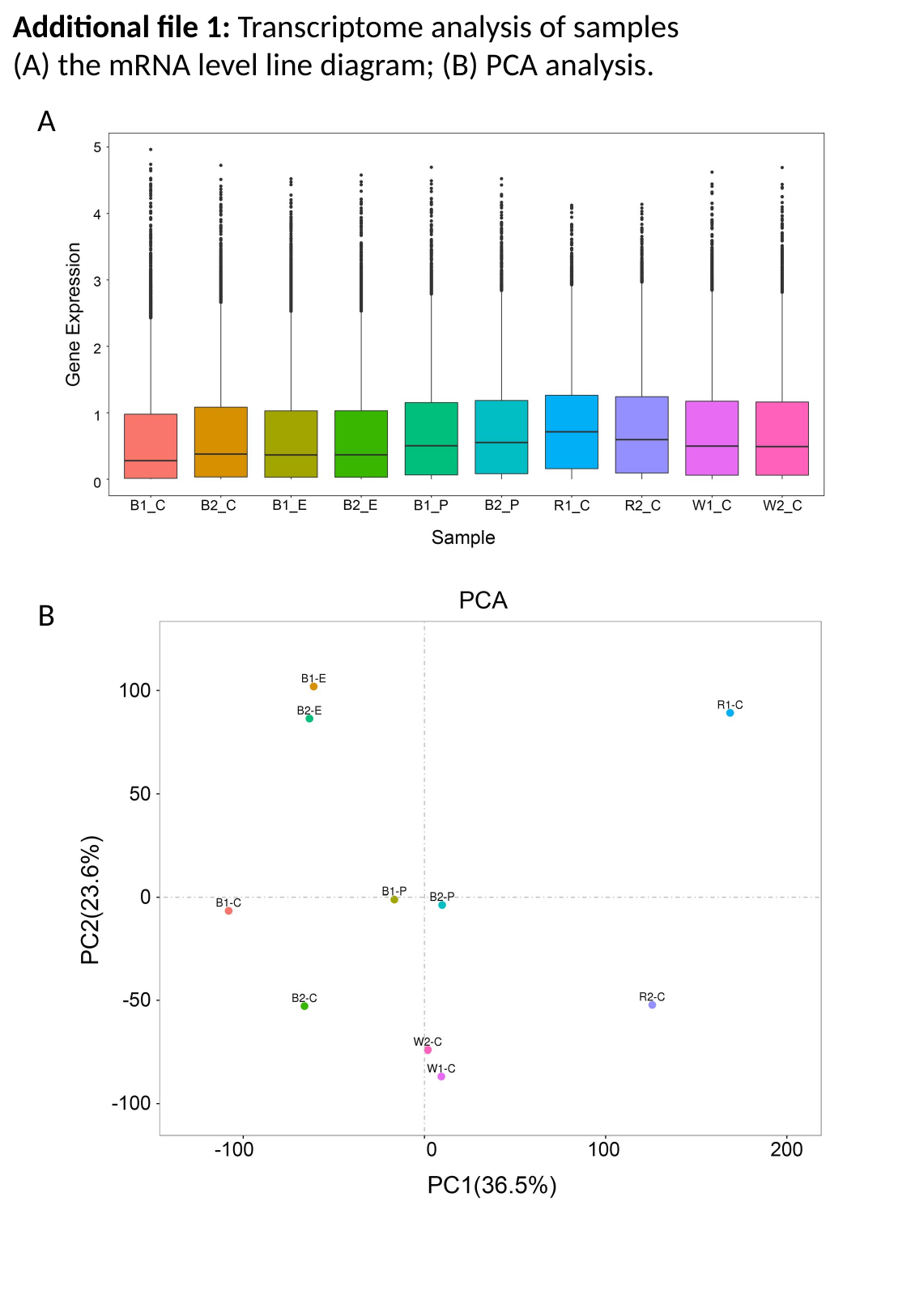

Additional file 1: Transcriptome analysis of samples
(A) the mRNA level line diagram; (B) PCA analysis.
A
B
