# Supplementary material for: Transcriptome and Proteome Profiling of Different Colored Rice Reveals Physiological Dynamics Involved in the Flavonoid Pathway
Source: Int J Mol Sci. 2019 May 18;20(10):2463. doi: 10.3390/ijms20102463 (PMC6566916; doi:10.3390/ijms20102463)
Supplement: Supplementary file 1 [file ijms-20-02463-s001.zip › ijms-496936-proof done-supplementary/Figure S2.pptx]

## Slide 1
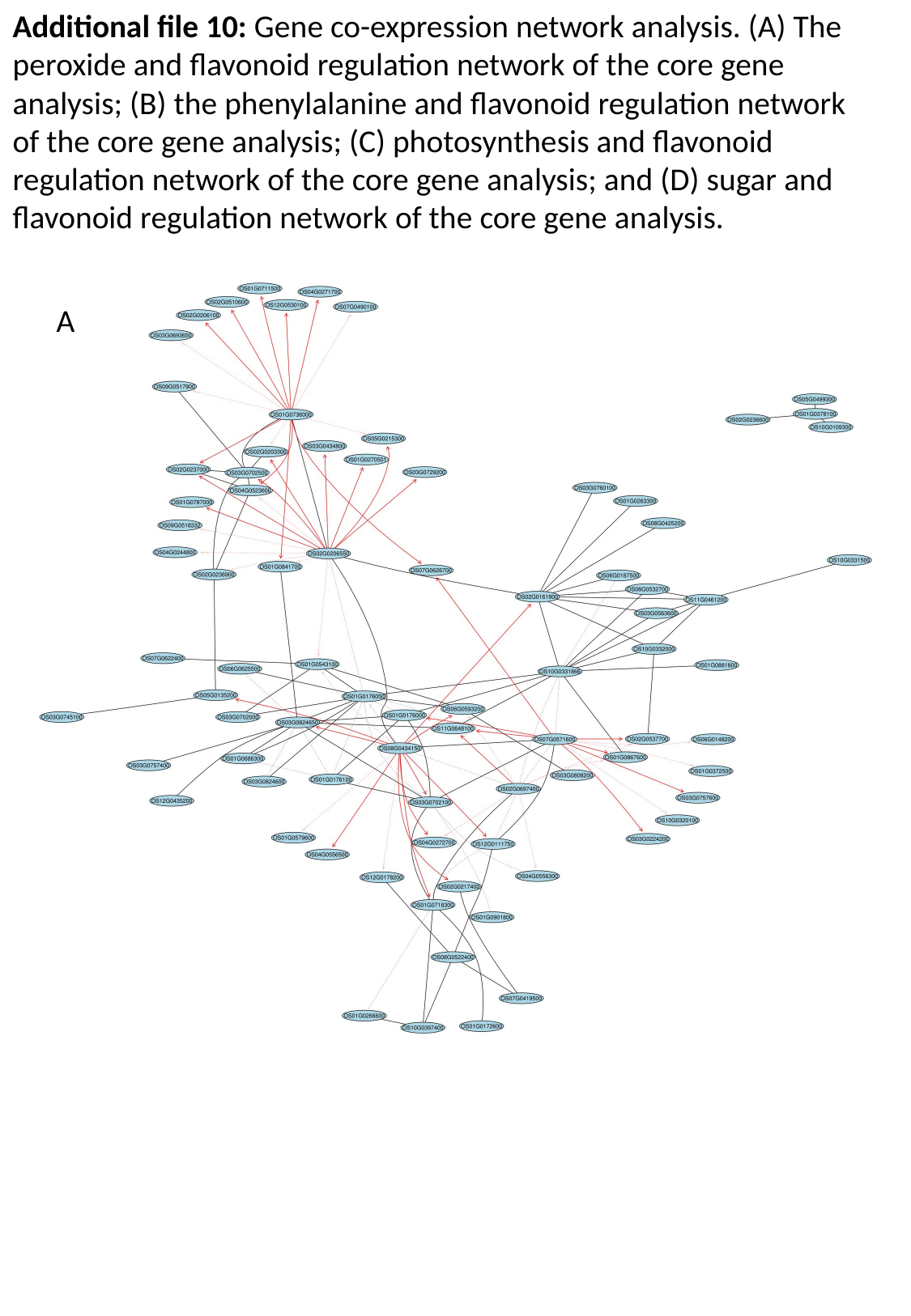

Additional file 10: Gene co-expression network analysis. (A) The peroxide and flavonoid regulation network of the core gene analysis; (B) the phenylalanine and flavonoid regulation network of the core gene analysis; (C) photosynthesis and flavonoid regulation network of the core gene analysis; and (D) sugar and flavonoid regulation network of the core gene analysis.
A

## Slide 2
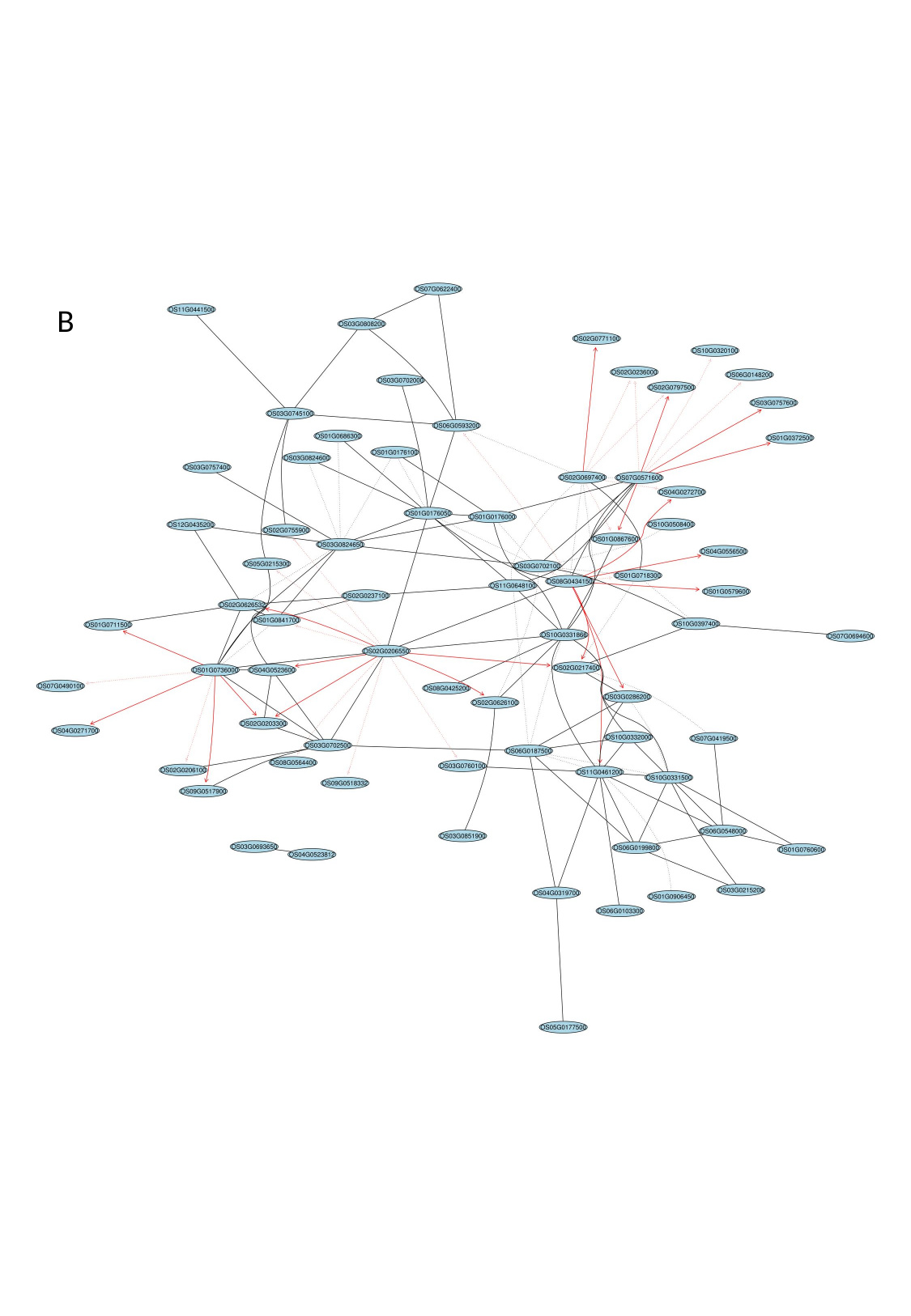

B

## Slide 3
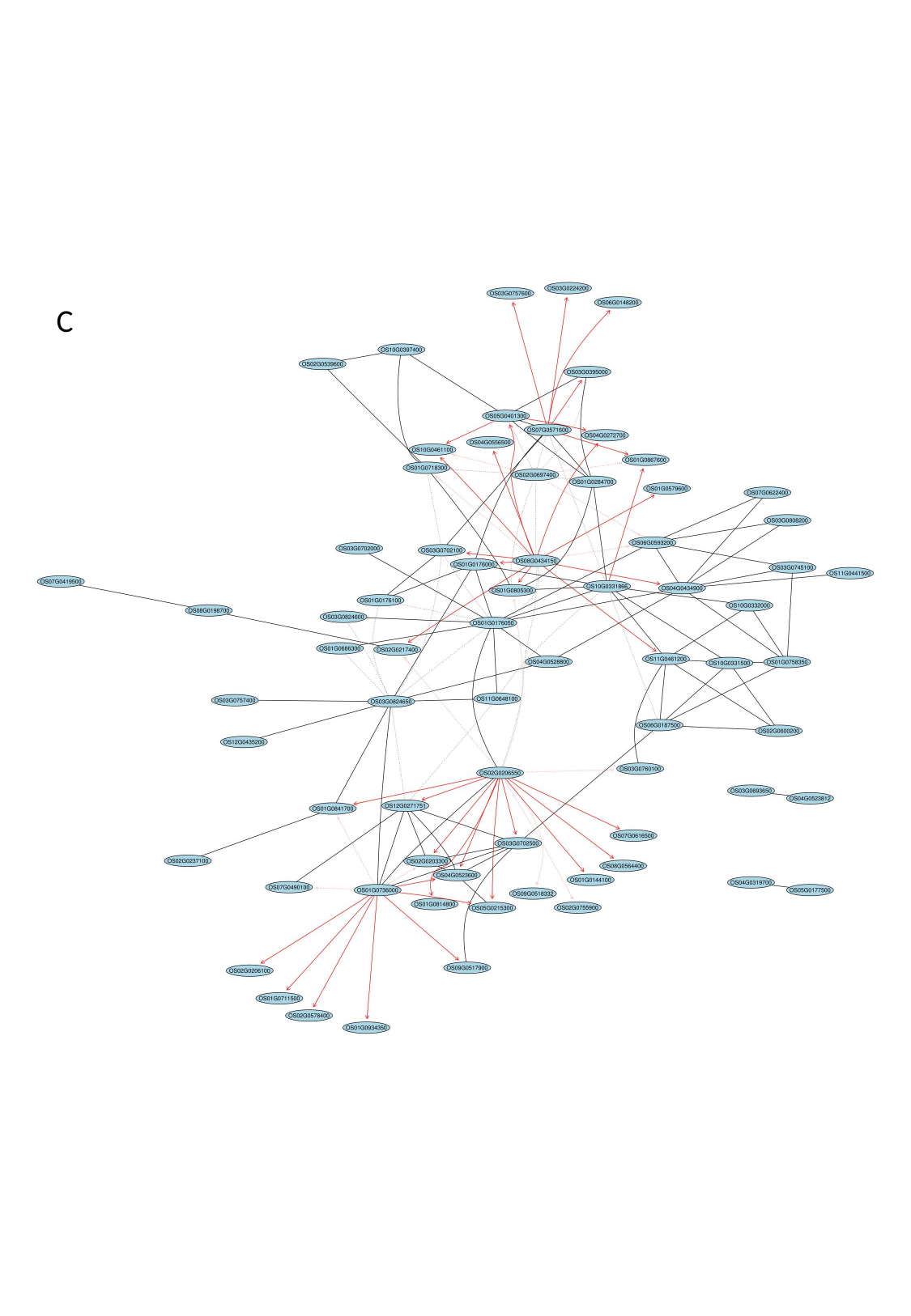

C

## Slide 4
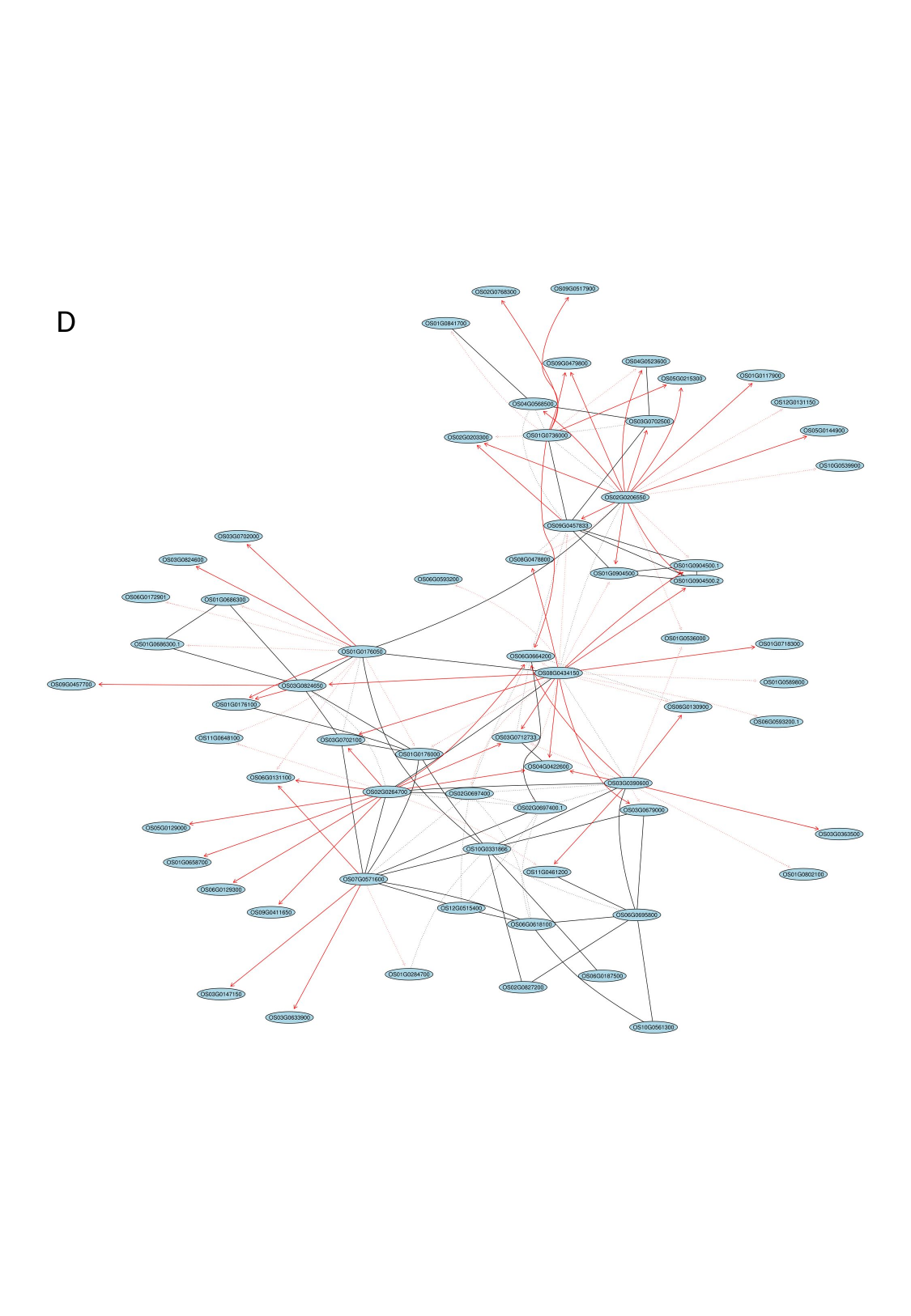

D
