# Supplementary material for: Transcriptome and Proteome Profiling of Different Colored Rice Reveals Physiological Dynamics Involved in the Flavonoid Pathway
Source: Int J Mol Sci. 2019 May 18;20(10):2463. doi: 10.3390/ijms20102463 (PMC6566916; doi:10.3390/ijms20102463)
Supplement: Supplementary file 1 [file ijms-20-02463-s001.zip › ijms-496936-proof done-supplementary/Table S2.pdf]

Additional file 2: Overview of transcriptome quality for the caryopses of the three rice cultivars and the pericarp and endosperm of black rice

Sample	Raw data	Clean data	Clean data ratio (%)	Aligned to genome	Mapping rate	Aligned to genes	Mapping rate	Expressed genes
B1-C	85875736	85420978	99.47	64702084	75.74%	62124682	72.73%	24762
B2-C	89091764	88498874	99.33	70522606	79.69%	64097704	72.43%	26303
R1-C	48246808	47874208	99.23	39091760	81.66%	34546578	72.16%	28104
R2-C	52317468	51873894	99.15	42023386	81.01%	36400264	70.17%	27208
W1-C	74381316	73901080	99.35	57071864	77.23%	52674924	71.28%	26816
W2-C	76523966	76125992	99.48	58727922	77.15%	54582540	71.70%	26975
B1-P	54674886	54366040	99.44	45308612	83.34%	40068992	73.70%	26731
B2-P	51549756	51241628	99.4	42536220	83.01%	37600336	73.38%	27004
B1-E	59018010	58584246	99.27	48564688	82.90%	44416134	75.82%	25448
B2-E	64909934	64469952	99.32	53982328	83.73%	49165754	76.26%	25554
