# Supplementary material for: Transcriptome and Proteome Profiling of Different Colored Rice Reveals Physiological Dynamics Involved in the Flavonoid Pathway
Source: Int J Mol Sci. 2019 May 18;20(10):2463. doi: 10.3390/ijms20102463 (PMC6566916; doi:10.3390/ijms20102463)
Supplement: Supplementary file 1 [file ijms-20-02463-s001.zip › ijms-496936-proof done-supplementary/Table S1.docx]

| **Gene/ Protein ID** | **Mean RatioB-C-VS-R-C** | **Mea RatioB-C-VS-W-C** | **Mean RatioR-C-VS-W-C** | **log2FC(B-C/R-C)** | **log2FC(B-C/W-C)** | **log2FC(R-C/W-C)** | **Description** |
| --- | --- | --- | --- | --- | --- | --- | --- |
| OS01G0850900 | - | 1.35 | 1.44 | - | - | 2.930368899 | heme-binding-like protein At3g10130, chloroplastic |
| OS08G0434100 | - | - | 0.83 | −5.040311748 | -3.361280923 | - | extracellular ribonuclease LE |
| OS10G0320100 | - | 0.66 | 0.56 | - | 2.681785408 | 3.706832427 | flavonoid 3'-monooxygenase |
| OS01G0638000 | 1.68 | 1.46 | 0.87 | −5.070762648 | - | - | anthocyanin 3'-O-beta-glucosyltransferase |
| OS11G0530600 | 3.86 | 6.95 | 1.57 | - | 4.641474522 | 6.273915733 | - |
| OS01G0372500 | 3.48 | 3.38 | - | 6.196397213 | 6.63840176 | - | leucoanthocyanidin dioxygenase |
| OS03G0819600 | 1.54 | 1.31 | - | - | −1.607364477 | - | chalcone--flavonone isomerase |
| OS06G0256500 | - | - | 0.78 | - | −1.379661054 | - | glucose-6-phosphate isomerase, cytosolic B |

**Table S1.** Significantly different genes based on fold-change levels for the three rice cultivars
