# Supplementary material for: Transcriptome and Proteome Profiling of Different Colored Rice Reveals Physiological Dynamics Involved in the Flavonoid Pathway
Source: Int J Mol Sci. 2019 May 18;20(10):2463. doi: 10.3390/ijms20102463 (PMC6566916; doi:10.3390/ijms20102463)
Supplement: Supplementary file 1 [file ijms-20-02463-s001.zip › ijms-496936-proof done-supplementary/Table S11.pdf]

Additional file 13: List of RT-PCR primers for flavonoid biosynthesis pathway genes

Gene	Primer-F	Primer-R	Product size
GAPDH	AAGCCAGCATCCTATGATCAGAT	CGTAACCCAGAATACCCTTG	144
Os04g0103900	CACATGTCGGACGAGTTCCT	GAGATTCTGGCGGACGTTGA	41
Os03g0289800	CATCGGCGACCAGATACAGG	TCCGCGGGTTGTAGAAGAAG	77
Os04g0630400	ACTTGGAGGATGAAGGCAGC	TGTGGCTCTTGTCGTACACC	51
Os03g0367101	CAACATCAGCGACTTCGTGC	TGGTGATGAGGACGTCGAAC	61
Os03g0184550	GAGGTTCAATGGGCGTTTA	TTTCACCTTCTCTGCCTTTC	83
Os11g0116300	GGTGATCAAGGAGATCAAAGG	CATCCTCCTCGTACTTGTCTA	54
Os01g0372500	GAGTGGGAGGACTACCTGTTC	ATGGCGAGCAGCTTGGAT	108
Os10g0536400	TATCCTCTCCACCAGTTCG	CCTGAACCCTAGCTCTCTTA	64
Os04g0630300	AAATACCCGCACCACAAC	CATGAACTCGAACCCTTCAC	73
Os04g0630600	GCTGTTGGAGAAGGGATATG	AGTCCTTGAGATGGGAGTT	42
Os04g0630800	CGACTACCTGAGAACAGAGA	GGCTCATGTTGTTCTCCTC	65
Os07g0601000	CATCGAGATCAGGCTCAAAG	CTCCACTCCATACCTGTACT	64
Os06g0651100	CTCGACCTCCTCAAAGAAAC	TCCAGTATCTCCTCCATTCC	68
Os01g0832600	CATCTCGCTCGCACTCTTCT	CGAAGGTCATCGGACGGTAG	71
Os04g0630900	GGGCTGATGAAAATGACGGC	AGACACCATACGCCTGCAAA	41
Os07g0598000	TGAGGGTGGGAGCTGATACA	TCCCATTCTCTTGGCGCATT	68
Os02g0767300	ATCAACTTCTACCCGCCGTG	GTACCAGTGGCCATCCTTGA	96
Os06g0683100	CAGCTCCTCCTCTCACCAAT	AAGGAGGGTAGTGCAAGGA	183
Os03g0184600	GCACCCCTATTCCATGTACCT	AGTTTCTTGGACTGTAGCCC	140
Os11g0566800	GCTACAACGAGGCATTCTGG	CCGACCTCCTCTTCATCTCC	141
Os03g0819600	GTGTGAGGGGAGTGGAGATC	CGTCACCTTGTCCGAGTACT	197
Os12g0115700	TCACCGTCAGTAAGCCGTTA	ACAAGCTCCTCTGCCTTCTT	121
Os04g0667200	GAAGTAAGGGTGGTGGCTCT	TGTGCTGCCAGACCTATTGA	125
Os08g0480200	CGGCGATCAGTTGGAGATTG	CCGTCTTCTCGTTGATGCTG	197
Os06g0162500	ACAGCGACAGCATGAAAG	GTTGTGGGCAAGGAGAATA	107
Os04g0630100	CGACAGTCAGAAACCCCGAA	CGACGAGGAAGGCGTAATC	110
Os06g0626700	CTGGGTCGTCTTCTGCGAG	TTGCACGTGCTGCTTGAATG	83
